# Deconstructing major depressive episodes across unipolar and bipolar depression by severity and duration: a cross-diagnostic cluster analysis on a large, international, observational study

**DOI:** 10.1038/s41398-020-00922-2

**Published:** 2020-07-19

**Authors:** Filippo Corponi, Gerard Anmella, Isabella Pacchiarotti, Ludovic Samalin, Norma Verdolini, Dina Popovic, Jean-Michel Azorin, Jules Angst, Charles L. Bowden, Sergey Mosolov, Allan H. Young, Giulio Perugi, Eduard Vieta, Andrea Murru

**Affiliations:** 1 Department of Biomedical and Neuromotor Sciences, University of Bologna, Bologna, Italy; 2 Bipolar and Depressive Disorders Unit, Institute of Neuroscience, Hospital Clínic, University of Barcelona, IDIBAPS, CIBERSAM, Barcelona, Catalonia Spain; 3 Biomedical Research Networking Center for Mental Health (CIBERSAM), Barcelona, Spain; 4 August Pi I Sunyer Biomedical Research Institute (IDIBAPS), Barcelona, Spain; 5 Psychiatry B, Chaim Sheba Medical Center, Ramat-Gan, Israel; 6 Department of Psychiatry, Sainte Marguerite Hospital, Marseille, France; 7 Department of Psychiatry, University of Zurich, Zurich, Switzerland; 8 Department of Psychiatry, University of Texas Health Science Center, San Antonio, TX USA; 9 Department for Therapy of Mental Disorders, Moscow Research Institute of Psychiatry, Moscow, Russia; 10 Department of Psychological Medicine, Institute of Psychiatry, Psychology & Neuroscience, King’s College London, Centre for Affective Disorders, London, UK; 11 Clinica Psichiatrica, University of Pisa, Pisa, Italy

**Keywords:** Depression, Bipolar disorder

## Abstract

A cross-diagnostic, post-hoc analysis of the BRIDGE-II-MIX study was performed to investigate how unipolar and bipolar patients suffering from an acute major depressive episode (MDE) cluster according to severity and duration. Duration of index episode, Clinical Global Impression-Bipolar Version-Depression (CGI-BP-D) and Global Assessment of Functioning (GAF) were used as clustering variables. MANOVA and post-hoc ANOVAs examined between-group differences in clustering variables. A stepwise backward regression model explored the relationship with the 56 clinical-demographic variables available. Agglomerative hierarchical clustering with two clusters was shown as the best fit and separated the study population (*n* = 2314) into 65.73% (Cluster 1 (C1)) and 34.26% (Cluster 2 (C2)). MANOVA showed a significant main effect for cluster group (*p* < 0.001) but ANOVA revealed that significant between-group differences were restricted to CGI-BP-D (*p* < 0.001) and GAF (*p* < 0.001), showing greater severity in C2. Psychotic features and a minimum of three DSM-5 criteria for mixed features (DSM-5-3C) had the strongest association with C2, that with greater disease burden, while non-mixed depression in bipolar disorder (BD) type II had negative association. Mixed affect defined as DSM-5-3C associates with greater acute severity and overall impairment, independently of the diagnosis of bipolar or unipolar depression. In this study a pure, non-mixed depression in BD type II significantly associates with lesser burden of clinical and functional severity. The lack of association for less restrictive, researched-based definitions of mixed features underlines DSM-5-3C specificity. If confirmed in further prospective studies, these findings would warrant major revisions of treatment algorithms for both unipolar and bipolar depression.

## Introduction

*The Diagnostic and Statistical Manual of Mental Disorders*, Fifth Edition (DSM-5) defines a major depressive episode (MDE) as a syndromic category wherein five (or more) symptoms are present and result in a clear-cut worsening of previous functioning during a minimum of 2 weeks^1^. DSM-5 construes MDE as unidimensional, aiming at an unachieved diagnostic reliability^2^, along with providing a number of specifiers defined as discrete entities. Thus, MDE is a transdiagnostic nosographic construct straddling major depressive disorder (MDD) and bipolar disorder (BD). The commonly used definitions of unipolar and bipolar depression represent an attempt to overcome this pitfall. Unipolar and bipolar depressions both impose a large burden for healthcare systems^3^. While as of today no clinical hallmark or biomarker can definitely differentiate a MDE as belonging to either MDD or BD^4^, depressions in MDD and BD probably do represent distinct conditions, with only partially overlapping genetic underpinnings^5^ and different therapeutic management^6–8^. Despite (hypo)mania being the earmark of BD, depression is generally the predominant mood state, representing the most prevalent polarity at illness onset and greatest contribution to psychosocial disability^9^.

As opposed to the plethora of specifiers included in the DSM-5, a model mapping gradations of severity and duration might capture much of the heterogeneity in depressive burden^10,11^. The inclusion of specifiers in the DSM-5 tried to bridge the need to assess severity and duration whilst keeping a categorical framework^12^. Illness severity can be conceptualized in terms of symptoms’ intensity and global functioning^13^. However, defining depression severity as a direct result of symptoms presence and intensity alone may be unjustified, due to the intrinsic heterogeneity of the depressive symptom as a clinical construct, and their different impact on the overall impairment that a patient may experience^14^. Along with the intensity of symptoms, their duration is a major element, which must be considered to perform a diagnosis of MDE^1^. Past studies link longer duration of index episode with greater severity^15–19^, higher comorbidities’ burden (i.e., dysthymia and anxiety), suicidal behavior^19^, and lower probability of recovery,^20^ furthermore, duration might not be related to the index episode being a recurrent or first-onset episode^19^.

The BDs: Improving Diagnosis, Guidance and Education-mixed features (BRIDGE-II-MIX) study was a large, multinational, cross-sectional study, which enrolled 2811 adults aged 18 years and older with MDE according to *Diagnostic and Statistical Manual of Mental Disorders*, Fourth Edition Text Revision (DSM-4-TR) diagnostic criteria at the time of the consultation and applied a descriptive, bottom-up approach with the primary aim of detecting mixed symptoms among such patients^21^. Several subanalyses have investigated the effects of comorbidity^22–24^, recurrence^25,26^, specific symptoms^27–31^, and treatment response^32,33^ in patients with MDE from this data set, but none looked into the impact of severity and duration.

Cluster analysis is a statistical technique that identifies subgroups in wider multidimensional or heterogeneous data, which application to multifaceted diseases, such as major depression, could help dissect disease heterogeneity, advancing diagnostic criteria, and improving treatment plans^34–36^.

Thus, the aim of this post-hoc study was to determine cross-diagnostic clinical clusters based on depressive burden, i.e., index episode’s clinical severity, and duration, within an acutely depressed population of unipolar and bipolar patients.

## Method

### Sample and assessment

The BRIDGE-II-MIX study was a multicenter, international, non-interventional, cross-sectional study conducted between June 2009 and July 2010 and involving 239 hospital based or community psychiatrists from eight countries across three continents, i.e. Bulgaria, Egypt, Morocco, the Netherlands, Portugal, Russia, Spain, and Turkey. The study enrolled 2811 adults (aged 18 years or older), experiencing an acute MDE according to DSM-IV-TR criteria (APA, 2000). In a single consultation, psychiatrist completed a structured case report for each patients, including inclusion criteria, sociodemographic variables, psychiatric medical history and features of the depressive episode, including DSM criteria for BD, previous response to antidepressants, psychiatric comorbidity and current treatment^21,27^. According to DSM-IV-TR, 735 patients (26.15%) fulfilled criteria for BD, of whom 400 patients (14.23%) met criteria for bipolar I disorder and 335 patients (11.92%) for bipolar-II disorder. On the other hand, 2076 patients (73.85%) met a DSM-IV-TR diagnosis of MDD, of whom 691 (24.58%) were at their first MDE.

The primary objective of the BRIDGE-II-MIX study was to establish the frequency of depressive mixed states by analyzing all the relevant symptoms of either pole. After the publication of DSM-5, this was post-hoc defined as (1) the proportion of patients fulfilling DSM-5 criteria for MDE with mixed features (American Psychiatric Association, 2013), and (2) research-based diagnostic criteria for mixed states (RBDC). DSM-5 criteria require the presence for at least a week of an MDE and at least 3 of the following (nonoverlapping) hypomanic symptoms: (1) elevated, expansive mood, (2) inflated self-esteem or grandiosity, (3) more talkative than usual or pressure to keep talking, (4) flight of ideas or subjective experience that thoughts are racing, (5) increase in energy or goal-directed activity, (6) increased or excessive involvement in activities that have a high potential for painful consequences, and (7) decreased need for sleep. The BRIDGE-II-MIX study also adopted the definition of DSM-5 subthreshold criteria for MDE with mixed features, for which the presence of an MDE plus 2 nonoverlapping hypomanic symptoms are required. RBDC are defined by the presence of MDE plus 3 out of the following 14 hypomanic symptoms for at least a week: irritable mood, affective lability, distractibility, psychomotor agitation, impulsivity, aggression (verbal or physical), racing thoughts, more talkative/pressure to keep talking, hyperactivity, increased energy, risky behavior, grandiosity, elation, and hyper-sexuality. The proportion of patients fulfilling criteria for BD according to the DSM-4-TR and bipolarity specifier proposed by Angst et al.^37–39^ was also identified. The bipolarity specifier attributes a diagnosis of BD to patients who experienced an episode of elevated mood or irritable mood or increased activity with at least three of the symptoms listed under Criterion B of the DSM-4-TR, associated with at least one of the three following consequences: (1) unequivocal and observable change in functioning uncharacteristic of the person’s usual behavior, (2) marked impairment in social or occupational functioning observable by others or (3) requiring hospitalization or outpatient treatment. No minimum duration was required, and no exclusion criteria were applied.

The study was conducted according to the Declaration of Helsinki (Hong Kong Amendment; http://www.wma.net) and the Good Epidemiology Practice and the International Epidemiologic Association (IEA) European Federation (http://iea.web.org). Good Epidemiologic Practice (GEP)—IEA Guidelines were followed for proper conduct of epidemiologic research, as well as pertinent national, legal, and regulatory requirements. Written informed consent was obtained from each patient. In each country, the protocol was approved by the local ethics committee.

### Measures of depressive severity and duration

We took a complete cases approach, including only patients without missing observations. If missing data meets the Missing Completely at Random (MCAR) assumption, removal of observations with missing data is expected to produce unbiased estimates of means, variances, and regression weights (Allison, 2002). We used the Hawkins test of multivariate normality and heteroscedasticity, as implemented in the ‘MissMech’ R package^40,41^, to address this question. A nonsignificant *p* value from this test would indicate a lack of sufficient evidence to reject the null hypothesis that data are MCAR. For this post-hoc analysis, we defined our clustering variables as: (1) duration of index episode: Collected retrospectively and measured in days. (2) Depression severity: Assessed at the time of consultation using the item for depression of the CGI-BP (CGI-BP-D)^42^ which evaluates how severely ill on a scale from 1 (normal, not ill) to 7 (very severely ill) a patient is. (3) Functional impairment: estimated at the time of consultation using the Global Assessment of Functioning (GAF)^43^, which measures how much a person’s symptoms affect his or her day-to-day life on a scale of 0–100. In line with previous recommendations, we checked that the sample size to include in the cluster analysis was no <5 × 2^k^ (*k* = number of clustering variables)^42^.

### Statistical analysis

#### Data normalization and exploratory analyses

Each measure was z-transformed prior to being entered as independent variable in the cluster analysis. Clustering tendency, i.e., inherent grouping structure, was assessed using the Hopkins statistic which examines whether objects in a data set differ significantly from the assumption that they are uniformly distributed in the multidimensional space. A value close to 1 indicates highly clustered data, random data results in values around 0.5 while uniformly distributed data yield values close to 0^44,45^.

#### Cluster analysis

Optimal clustering algorithm and number of clusters were determined using the ‘clValid’ R package^46^, which allows for simultaneous comparison of multiple clustering algorithms and numbers of clusters in terms of validation measures. We tested for the presence of two to six clusters and we implemented three clustering methods: (1) k-means (2) k-medoids or partitioning around medoids (PAM) and (3) agglomerative hierarchical clustering^47,48^. The validation measures used to compare different clustering solutions comprised internal and stability criteria. Internal criteria were calculated as connectivity, silhouette width and Dunn index. Stability criteria upon removal of each variable were: APN (average proportion of non-overlap); AD (average distance); ADM (average distance between means between cluster centers); FOM (figure of merit).

Cluster group means for all measures of depressive burden, i.e., index episode duration, CGI-BP-D, and GAF, were compared using MANOVA, each measure of depressive burden was than examined separately using ANOVA to assess individual contribution. Subsequently, a stepwise backward logistic regression model was used to identify the predictive value on cluster membership of the 56 relevant clinical-demographic features available from the BRIDGE-II-MIX study. The Akaike information criterion (AIC) was used for stepwise backward variable selection. AIC performs feature selection striking a balance between goodness of fit and overfitting. Stepwise backward logistic regression was carried out using the ‘MASS’ R package^49^. Odds ratios (ORs) with 95% confidence intervals (CIs) were calculated for the observed associations.

All statistical analyses were performed using R Statistical Software (Foundation for Statistical Computing, Vienna, Austria).

## Results

### Cluster analysis

Out of the 2811 patients considered, after screening for missing values 2314 complete cases were included in the analysis. Missing data met the MCAR assumption, thus supporting listwise deletion as an appropriate approach. Hopkins index of 0.15 suggested inherent grouping structure. All internal validation measures and 2 out of 4 stability validation criteria favored agglomerative hierarchical clustering with 2 clusters as optimal clustering fit to the data set (Supplementary Fig. 1). Dendrogram produced from Ward’s clustering (Supplementary Fig. 2) was cut to produce two clusters and subjects were assigned cluster membership accordingly.

C1 and C2 comprised 65.73% (*n* = 1521) and 34.26% (*n* = 793) of the study population respectively. Using Pillai’s trace, MANOVA showed a significant main effect for cluster group (*V* = 0.35, *F*_3,2312_ = 432, *p* < 0.001) but, as revealed by ANOVA, significant between-group differences were restricted to CGI-BP-D (*F*_1,2312_ = 190, *p* < 0.001) and GAF (*F*_1,2312_ = 1297, *p* < 0.001), both indicating greater disease severity in C2. Duration of index episode did not significantly separate the two clusters (*F*_1,2312_ = 0.34, *p* = 0.56).

### Stepwise AIC backward logistic regression

**Fig. 1:**
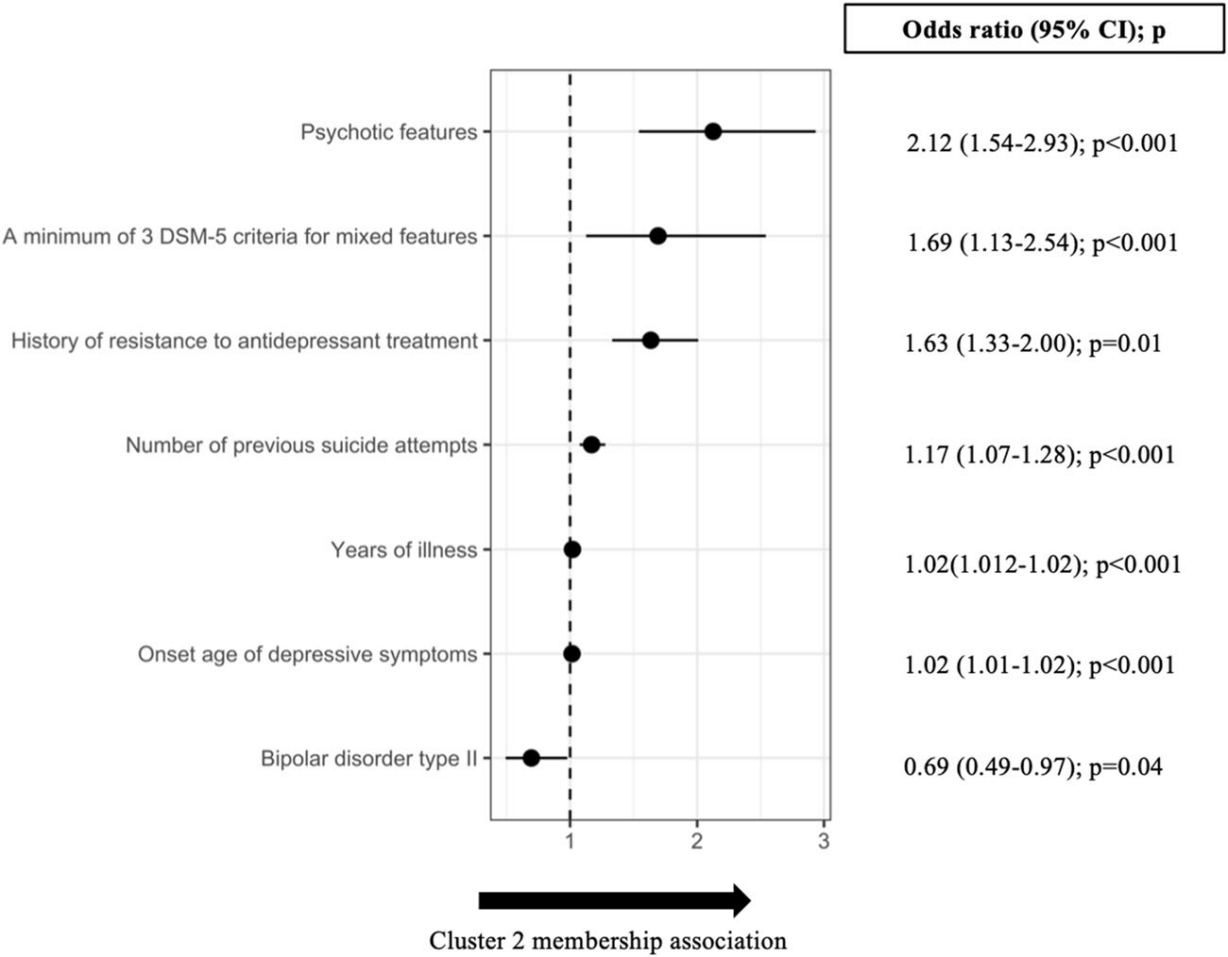
Odds ratios (ORs) with 95% confidence intervals (95% CIs) are shown for the 7 variables with *p* value < 0.05 out of the 19 variables in the final stepwise Akaike information criterion (AIC) backward logistic regression model. ORs are expressed with respect to Cluster 2, i.e., the cluster displaying grater depressive burden.

Starting with the full model including all 56 clinical-demographic independent variables (Table 1), stepwise backward selection of independent variables reached AIC optimization at 19 variables (Table 2): 34 variables were thus removed from the full model. The final 19 variables model showed significantly better fit than the null model (*χ*^2^ = 148, df = 19, *p* < 0.001). In the final model, 7 out of 19 of the variables retained were significantly associated with cluster membership. Relative ORs with 95% CIs are shown in Fig. 1. Psychotic features (OR = 2.12, 95% CI = 1.54; 2.93) was the variable most strongly associated with C2 membership, followed by a minimum of three DSM-5 criteria for mixed features (OR = 1.69, 95% CI = 1.13; 2.54) and history of antidepressant resistance (OR = 1.63, 95% CI = 1.33; 2.00). Years of illness (OR = 1.02, 95% CI = 1.01–1.02) and onset age of depressive symptoms (OR = 1.02, 95% CI = 1.01–1.02) had only a tiny positive association. On the contrary, BD type II was negatively associated with C2 membership (OR = 0.69, 95% CI = 0.49; 0.97).

**Table 1:** The 56 clinical-demographic variables available from the BRIDGE-II-MIX study and explored in the stepwise backward logistic regression model.

	Cluster 1 (*n* = 1521)		Cluster 2 (*n* = 793)	
	*n*	%	*n*	%
Categorical variables (YES)				
Female gender	1057	69.49	536	67.59
Inpatient status	366	24.06	244	30.76
Family history of bipolar disorder among first degree relatives	230	15.12	120	15.13
Diagnosis				
Bipolar disorder type I	200	13.21	142	17.9
Bipolar disorder type II	190	12.42	95	11.97
First major depressive episode	350	23.01	168	21.12
Major depressive disorder	781	51.34	388	48.92
Concurrent symptoms experienced most of the time during the past week				
Anxiety	1211	79.61	623	78.56
Hyperphagia	218	14.33	114	14.37
Hypersomnia	244	16.04	120	15.13
Hypo-sexuality	935	61.47	533	67.21
Insomnia	1084	71.26	580	73.13
Leaden paralysis	364	23.93	222	27.99
Memory problems	935	60.81	486	61.28
Mood reactivity	646	42.47	317	39.97
Panic attacks	225	14.79	133	16.77
Psychotic features	85	5.58	99	12.48
Reduced appetite	905	59.50	514	64.82
Research-Based Diagnostic Criteria (RBDC) for mixed states				
Distractibility	371	24.39	214	26.98
Elation	65	4.27	47	5.92
Grandiosity	46	3.02	49	6.17
Hyperactivity	110	7.23	82	10.34
Hyper-sexuality	36	2.36	27	3.40
Impulsivity	214	14.06	118	14.88
Increased energy	90	5.91	74	9.31
Irritable mood	489	32.14	259	32.66
Mood lability	464	30.50	239	30.14
More talkative/Pressure to talk	168	11.04	106	13.36
Psychomotor agitation	224	14.72	141	17.78
Racing thoughts	160	10.51	110	13.87
Risky behavior	94	6.18	71	8.95
Verbal or physical aggressivity	194	12.75	122	15.38
Past response to antidepressant treatment				
History of (hypo)manic switches ensuing antidepressant treatment	260	17.09	163	20.55
History of resistance to antidepressant treatment	359	23.60	278	35.05
History of mood lability ensuing antidepressant treatment	440	28.92	269	33.92
History of irritability ensuing antidepressant treatment	374	24.59	239	30.13
Mixed affect definitions				
A minimum of 2 DSM-5 criteria for mixed features	217	14.26	147	18.53
A minimum of 3 DSM-5 criteria for mixed features	98	6.44	93	11.73
A minimum of 2 intradepression (hypo)manic symptoms	588	38.65	326	41.1
A minimum of 3 intradepression (hypo)manic symptoms	420	27.61	247	31.14
Comorbidities				
Alcohol abuse	97	6.37	53	6.68
Borderline personality disorder	90	5.91	67	8.44
Generalized anxiety disorder	264	17.35	141	17.78
Obsessive-compulsive disorder	73	4.79	43	5.42
Panic disorder	148	9.73	89	11.22
Social phobia	106	6.96	70	8.82
Substance abuse	39	2.56	26	3.27
	Mean	SD	Mean	SD
Numeric variables				
Days, if any, with (hypo)mania in the last year	12.1	31.9	14.7	33
Days with depression in the last year	85.7	65.5	89	58.3
Number of mood (depressive and/or (hypo)manic) episodes in the last year	2.17	4.35	2.70	5.62
Number of previous depressive episodes	4.58	5.7	5.47	6.89
Number of previous hospitalizations	1.59	3.26	2.31	5
Number of previous suicide attempts	0.32	0.91	0.65	2.3
Onset age of depressive symptoms	34.9	12.5	36.1	12.7
Total number of Research-Based Diagnostic Criteria (RBDC) for mixed states	1.79	2.57	2.09	2.99
Years of illness	7.79	9.38	9.44	10.1

Values across Cluster 1 and Cluster 2 are shown.

**Table 2:** The 19 variables selected in the final logistic regression model.

Variable	Coefficient	SE	*z* value	*p* value
A minimum of 3 DSM-5 criteria for mixed features	0.53	0.21	2.54	0.01*
Bipolar disorder type II	−0.36	0.17	−2.08	0.04*
First major depressive episode	0.04	0.17	0.25	0.80
Grandiosity	0.40	0.26	1.54	0.12
History of resistance to antidepressant treatment	0.49	0.10	4.69	<0.001*
Hypo-sexuality	0.19	0.10	1.90	0.06
Impulsivity	−0.28	0.16	−1.72	0.09
Major depressive disorder	−0.23	0.14	−1.72	0.09
Memory problems	−0.18	0.10	−1.84	0.07
Mood lability	−0.23	0.12	−1.93	0.05
Mood reactivity	−0.15	0.10	−1.54	0.12
Number of mood (depressive and/or (hypo)manic) episodes in the last year	0.02	0.01	1.62	0.10
Number of previous suicide attempts	0.16	0.04	3.57	<0.001*
Onset age of depressive symptoms	0.02	0.00	4.02	<0.001*
Psychotic features	0.75	0.16	4.60	<0.001*
Racing thoughts	0.23	0.16	1.43	0.15
Reduced appetite	0.19	0.10	1.93	0.05
Verbal or physical aggressivity	0.26	0.15	1.72	0.09
Years of illness from onset	0.02	0.01	3.42	<0.001*

The Akaike information criterion (AIC) was used for stepwise backward variable selection, starting from the full model with 56 variables. Cluster membership (the dependent variable) was coded as 1 for Cluster 2 (positive case) and 0 for Cluster 1 (negative case).

*p values < 0.05.

## Discussion

This post-hoc analysis of the BRIDGE-II-MIX study aimed at clustering a sample of acutely depressed patients according to measures of depressive burden, i.e., symptom intensity, functional impairment, and duration. Our analysis identified two clusters, showing little (C1) and great (C2) severity. The great severity cluster C2 included ~1 out of 3 patients displaying a significantly greater depressive burden as defined by CGI-BP-D and GAF, whereas the duration of the index episode did not significantly contribute to clusters’ separation. Among other possible explanations, such as differences in latency to access to mental health-related services, the cross-sectional design of the study may account for the lack of clusters’ separation by longer illness duration, since records for index episode’s duration did not cover the entire course of the episode. Interestingly, the cluster including the most severe patients has BD less represented (proportionally) as diagnosis subgroup in comparison to the other cluster.

The regression model revealed that psychotic features had the largest OR for positive association with C2 membership (Fig. 1). The link between psychosis and depression severity is indeed long-established^50,51^, since the presence of psychotic features underpins cognitive and processing impairments across multiple severe mental disorders, irrespective of the clinical diagnosis^52^.

A key finding of our study is that the clinical presentation with mixed features according to DSM-5 criteria represents the second strongest association with the C2. Strengthening this result, pure, non-mixed presentation in acute bipolar-II depression represents the unique significant negative association with illness severity. Thus, the absence of mixed features seems a protective factor for illness severity and interestingly this was not influenced by possible treatment differences between BD types I and II. To the best of our knowledge, this finding has no precedent in scientific literature. Despite the limitations further disclosed, should it be confirmed by future, prospective evidence, this finding could have an impact on the methodology of studies designs requested by regulatory agencies. In fact, these results go against the common depiction of a severity progression spanning from unipolar depression, to BD-II and BD-I.

Overall, mixed features might be considered as a marker of severity both in research and clinical settings, outlining a functionally compromised group of patients irrespectively of the diagnosis^53–55^. Yet, according to our results mixed states do not invariantly represent a marker of clinical severity. In fact, the statistical contribution to C2 in our study was observed for mixed affect defined as the presence of at least three DSM-5 symptoms, but not as determined according to other less conservative definitions, i.e., RBDC criteria for mixed features and a minimum of two DSM-5 symptoms. This could be explained by the fact that RBDC criteria have a higher sensitivity for detection of mixed states^56^, but this does not directly translate into a more severe MDE, bearing doubtful acute prognostic implications. On the other hand, DSM-5 criteria, specifically a minimum of three symptoms^1^, seem to have higher specificity in diagnosing more severe mixed presentations, reflected by the statistical assignment to our C2.

Last, the C2 association in the regression model with previous suicide attempts and previous resistance to antidepressant treatments could be considered the hallmark of mixed presentation, as previously outlined in this very sample^33^ and previous studies on suicidal ideation and psychotic features marking more severely impaired MDE patients^57^. In particular, a history of previous resistance to antidepressant treatments bears important treatment implications. In fact, as suggested elsewhere, mixed-features depressions not responding to antidepressants might benefit from coupling a mood-stabilizing agent or even dropping the antidepressant altogether^58^.

Results coming from this post-hoc analysis should be balanced against some limitations. First, due to the cross-sectional design of the BRIDGE-II-MIX recall bias probably affected some of the variables’ definitions, and especially our definition of duration, whose esteem might also depend on external factors (e.g., latency of referral and psychiatric assessment). Also, the assessment of symptom severity and functioning may have been somewhat hampered by the use of the GAF as a functional outcome, as its anchor points rely also on symptoms severity. This might have contributed to the strong correlation between CGI and GAF scores in our sample. Also, concerns about inter-rater reliability have been raised in the past, but its validity and clinical usefulness on the assessment of symptom and function dimensions have been established^59^. A deeper assessment of the overall functioning of the patients should have accounted for different dimensions that would have allowed for an increased discriminant capacity^60^. Second, the participating centers were not randomly selected, which may have led to a bias through the inclusion of psychiatrists with a particular interest in mixed states. However, this could also represent a study strength as some expertise is needed to detect mixed states in MDE patients. Another limitation was the wide variation in the rates of hospitalized patients across countries, ranging from 1.0 to 57.8%, which could reflect economically driven policies on the use of hospitalization-based treatment rather than real differences in clinical practice or patients’ severity. The main strengths of the BRIDGE-II-Mix study include the large sample size and the wide range of care settings, both hospital and community, from eight countries across three continents. Furthermore, narrow exclusion criteria increase the generalizability of the findings. Lastly, it should be acknowledged that cluster analysis is a data mining technique for finding patterns in data and is exploratory in nature.

In conclusion, in this post-hoc cluster analysis on a large international sample of 2314 acutely depressed patients, approximately one third of the study population fitted into a significantly more severe cluster of patients, independently of the traditional, categorical diagnosis. Mixed states seem to challenge the traditional unipolar–bipolar dichotomy and bridge the gap between these two categories of mood disorders. Besides well-documented and straightforward associations, such as current psychotic symptoms, history of resistance to antidepressant treatment and a history of suicide attempts, mixed affect defined as the co-occurrence of a minimum of three DSM-5 criteria for mixed features was significantly associated to more severe acute depression, supporting the usefulness of this definition for mixed features as significantly relevant to depression severity.

According to our results, a BD diagnosis does not automatically imply a worse severity of depression. In contrast, mixed features according to the current nosology should be considered as an intrinsic marker of severity, independently of a bipolar/unipolar diagnosis, in acutely depressed patients. This points to a continuum of the mood spectrum as a unitary phenomenon.

## Supplementary information

Supplementary Figure 1

Supplementary Figure 2

## Data Availability

All R-code we developed for statistical modeling is available upon request.
