# Supplementary material for: Deconstructing major depressive episodes across unipolar and bipolar depression by severity and duration: a cross-diagnostic cluster analysis on a large, international, observational study
Source: Transl Psychiatry. 2020 Jul 19;10:241. doi: 10.1038/s41398-020-00922-2 (PMC7370235; doi:10.1038/s41398-020-00922-2)
Supplement: Supplementary file 1 — Supplementary Figure 1 [file 41398_2020_922_MOESM1_ESM.docx]

**Figure 1**. (a) Comparison of different clustering algorithms and number of clusters on internal and stability validation measures. (b) Best clustering algorithm and number of clusters according to each validation measure.

(a)

| Internal Validation | | | | | |
| --- | --- | --- | --- | --- | --- |
| Connectivity | | Silhouette Width | | Dunn | |
| 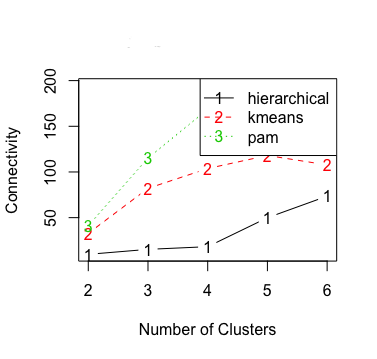 | | 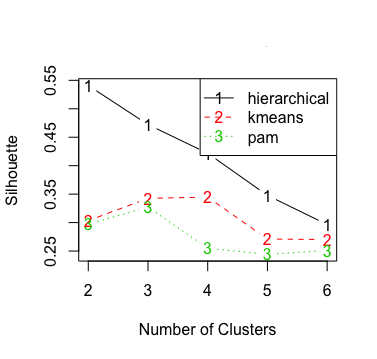 | | 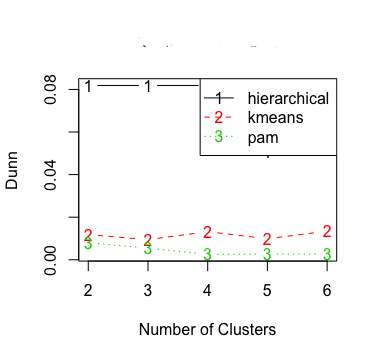 | |
| Stability Validation | | | | | |
| APN | AD | | ADM | | FOM |
| 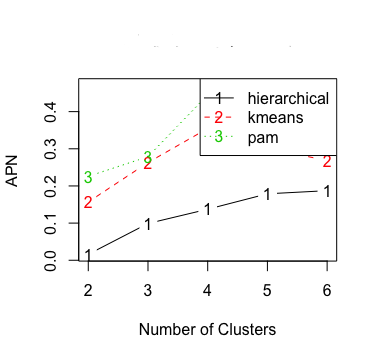 | 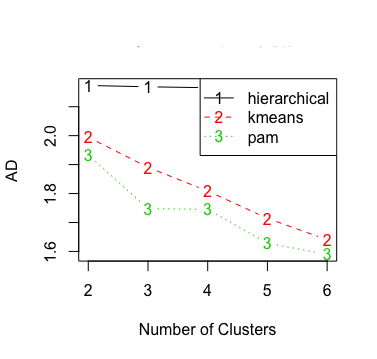 | | 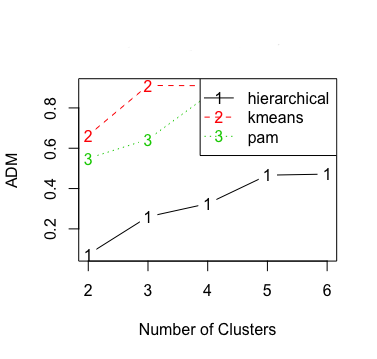 | | 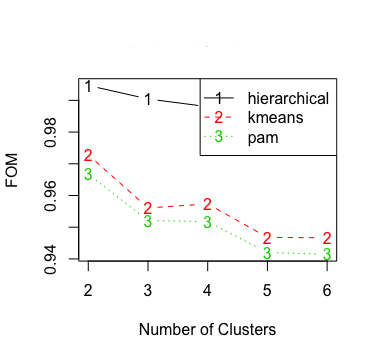 |

Connectivity, to what extent are individuals placed in the same cluster as the most similar individuals, should be minimized (scale = 0, ∞); Silhouette Width, overall average of average distance between individual and others in same cluster compared to different cluster, should be maximized (−1, 1); Dunn Index, smallest inter-cluster distance/largest intra-cluster distance, should be maximized (scale = 0, ∞); APN, average proportion of non-overlap or number of individuals not placed in same cluster when variable is removed, should be minimized (scale = 0,1); AD, average distance between individuals placed in same cluster when variable is removed, should be minimized (scale = 0, ∞); ADM, average distance between means between cluster centers for individuals placed in same cluster when variable is removed, should be minimized (scale = 0, ∞); FOM, figure of merit or average intra-cluster variance of the removed variable where clustering is based on remaining variables, should be minimized (scale = 0, ∞).

(b)

| Validation measure | Score | Clustering algorithm | Number of clusters |
| --- | --- | --- | --- |
| Connectivity | 9.85 | Agglomerative hierarchical | 2 |
| Silhouette width | 0.08 | Agglomerative hierarchical | 2 |
| Dunn | 0.54 | Agglomerative hierarchical | 2 |
| APN | 0.02 | Agglomerative hierarchical | 2 |
| AD | 1.59 | Partitioning around medoids | 6 |
| ADM | 0.07 | Agglomerative hierarchical | 2 |
| FOM | 0.94 | Partitioning around medoids | 6 |
