# Supplementary material for: Deconstructing major depressive episodes across unipolar and bipolar depression by severity and duration: a cross-diagnostic cluster analysis on a large, international, observational study
Source: Transl Psychiatry. 2020 Jul 19;10:241. doi: 10.1038/s41398-020-00922-2 (PMC7370235; doi:10.1038/s41398-020-00922-2)
Supplement: Supplementary file 2 — Supplementary Figure 2 [file 41398_2020_922_MOESM2_ESM.docx]

**Figure 2**. (a) Dendrogram - Ward’s method was used with squared Euclidean distance as a proximity measure. (b) Depression severity measures by cluster, multivariate analysis of variance (MANOVA), analysis of variance (ANOVA).

(a)


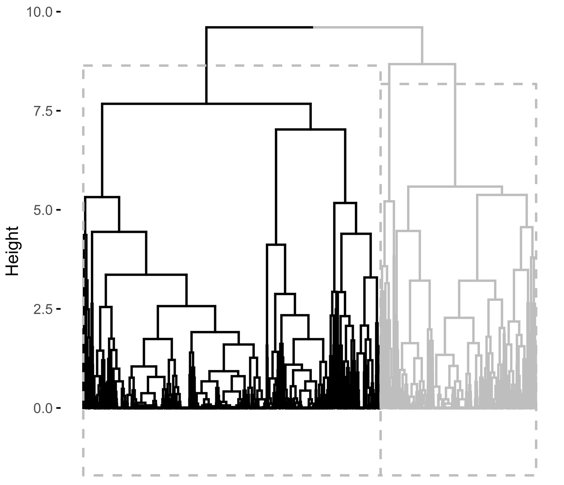


(b)

|  | Mean (±SD) | | MANOVA | ANOVA |
| --- | --- | --- | --- | --- |
|  | Cluster 1  n=1521 | Cluster 2  n=793 |  |  |
| Duration of current episode | 59.90 (±50.44) | 61.10 (±34.70) | V=0.35, F_3,2312_=432, p<0.001 | F_1,2312_=0.34, p>0.05 |
| CBI-BD-D | 4.27 (±0.77) | 4.82 (±1.11) |  | F_1,2312_=190, p<0.001 |
| GAF | 56.20 (±9.24) | 40.10 (±11.90) |  | F_1,2312_=1297, p<0.001 |
